# High Glucose Promotes CD36 Expression by Upregulating Peroxisome Proliferator-Activated Receptor *γ* Levels to Exacerbate Lipid Deposition in Renal Tubular Cells

**DOI:** 10.1155/2017/1414070

**Published:** 2017-04-12

**Authors:** Lei Feng, Chengwu Gu, Yanxia Li, Jiasui Huang

**Affiliations:** ^1^Graduate School, The Second Affiliated Hospital of Chongqing Medical University, Chongqing 400010, China; ^2^Hospital Infection Control Department, The Suining Central Hospital, Suining 629000, China

## Abstract

Diabetic kidney disease (DKD) appears to be closely related to lipid deposition in kidney. The aim of this study was to determine whether high glucose (HG) exacerbated lipid deposition by increasing CD36 expression via AKT-PPAR*γ* signaling pathway. Our results showed that HG activated AKT signaling pathway, followed by an increase in PPAR*γ* that induced CD36 overexpression, ultimately causing lipid deposition in HK-2 cells. We also found that inhibition of AKT-PPAR*γ* signaling pathway or knockdown of CD36 could reduce HG-induced lipid accumulation in HK-2 cells. These results indicated that AKT-PPAR*γ* signaling pathway mediated HG-induced lipid deposition by upregulating CD36 expression in HK-2 cells and that inhibition of AKT-PPAR*γ* signaling pathway had the potential beneficial effects of reducing lipid deposition in diabetic kidney.

## 1. Introduction

Diabetes mellitus (DM), which induces decreased expectancy and quality of life, is a group of systemic metabolic diseases characterized by hyperinsulinemia, chronic hyperglycemia, dyslipidemia, hypertension, inflammation, and proteinuria, with two major types being type 1 or type 2 [1, 2]. DKD, characterized by proteinuria, glomerulosclerosis, and decreased glomerular filtration rate, is the most common microvascular complications of DM and the major cause of end-stage renal failure, which seriously affects patient quality of life [3–6]. Up to now, accumulating evidences suggest that hyperglycemia plays a critical role in the pathogenesis of diabetic micro- and macrovascular complications, including DKD, neuropathy, retinopathy, and atherosclerosis [7–10]. As previously shown, hyperglycemia could increase reactive oxygen species (ROS) generation and induce oxidative stress in diabetes, exacerbating renal injury [11, 12]. Moreover, recent publications have suggested a close association between hyperglycemia and lipid deposition in diabetic kidney [13–15]. It is noted that renal lipid deposition of diabetes may play an essential role in DKD progression [16–19]. Although a correlation between hyperglycemia and lipid deposition in diabetic kidney has been confirmed in human studies and in multiple animal models, further research targeting the underlying molecular mechanisms of this relationship is required.

In several tissues, many of free fatty acids are taken up through transporters in cell membrane and some enter cells by simple diffusion. As a long-chain fatty acid transporter, the class B scavenger receptor CD36 is an 88-kDa transmembrane glycoprotein and clearly responsible for lipid deposition in several tissues [20, 21]. CD36 expression is markedly elevated in the HG-treated HK-2 cells and renal tubular cells in human diabetic kidneys [2, 22]. Furthermore, increased CD36 expression can mediate HG-induced epithelial to mesenchymal transition and apoptosis in HK-2 cells [22, 23]. We therefore hypothesize that HG induces renal lipid deposition by upregulating the expression of CD36. Although several studies have demonstrated that HG upregulates CD36 expression in renal cells, the exact mechanism remains unknown.

Peroxisome proliferator-activated receptor *γ* (PPAR*γ*), a ligand-activated transcription factor, can bind as a heterodimer with retinoid X receptor *α* to regulatory elements in responsive genes, which in turn activate the transcription of target genes including CD36. Several studies have demonstrated that increased PPAR*γ* can upregulate CD36 expression and function, while PPAR*γ* knockout can dramatically reduce CD36 expression and function in many different types of cells, including macrophages, hepatocytes, and kidney cells [24–27]. As a nuclear transcription factor, PPAR*γ* can be regulated by many factors and then up- or downregulate CD36 expression and function.

However, whether PPAR*γ*-mediated CD36 expression participates in HG-induced lipid accumulation in renal tubular cells has not been reported. As a genetic factor, PPAR*γ* plays an important role in modulating lipid metabolism, which can be regulated by various factors [28]. Previous studies have shown that phosphorylation of AKT also involves regulating lipid metabolism by activating downstream effectors in diabetic kidney [29]. The present study was performed to investigate whether HG enhanced CD36 expression by upregulating AKT-mediated PPAR*γ*, which exacerbated lipid deposition in renal tubular cells.

## 2. Materials and Methods

### 2.1. Cell Culture and Treatments

As the human renal tubule epithelial cells, the HK-2 cells (a gift from Dr. Xiong Z. Ruan) were cultured at 37°C in a 5% CO_2_ atmosphere, 95% air in no glucose Roswell Park Memorial Institute (RPMI) 1640 medium supplemented with 10% fetal bovine serum. All experiments were performed in the experimental media (0.2% bovine serum albumin + no glucose RPMI 1640). Before harvesting, the HK-2 cells were preconditioned in the experimental media for at least 12 hours and then stimulated with normal glucose (NG) (D-glucose 5.6 mM), NG plus LY294002 (15 uM), NG plus rosiglitazone (RSG, 5 uM), NG plus GW9662 (2.5 uM), high glucose (HG) (D-glucose 30 mM), HG plus rosiglitazone (RSG, 5 uM), or HG plus GW9662 (2.5 uM). Nontargeting control siRNA (NTC siRNA) or CD36 siRNA transfections of HK-2 cells were conducted in 24 well plates with Lipofectamine 2000 (Invitrogen, Carlsbad, CA) according to the manufacturer's instructions. The sequences for CD36 siRNA were (sense) 5′-GGCUGUGUUUGGAGGUAUUCUTT-3′ and (anti-sense) 5′-AGAAUACCUCCAAACACAGCCTT-3′. The sequences for NTC siRNA were (sense) 5′-UUCUUCGAACGUGUCACGUTT-3′ and (anti-sense) 5′-ACGUGACACGUUCGGAGAATT-3′. siRNA oligonucleotides were synthesized by Shanghai GenePharma, China. Reduction of CD36 levels was confirmed using western blotting. LY294002, an inhibitor of AKT phosphorylation, was purchased from Beyotime Institute of Biotechnology (Haimeng, China). Rosiglitazone (RSG) and GW9662 were acquired from Sigma-Aldrich (St. Louis, MO, USA). RSG is a highly selective PPAR*γ* agonist and GW9662 is a selective PPAR*γ* antagonist.

### 2.2. Western Blot Analysis

Total protein from HK-2 cells was extracted using RIPA buffer and then 30 ug of each sample protein was resolved by SDS-PAGE and transferred to polyvinylidene fluoride membranes (Millipore Corporation, Bedford, MA, USA). The membranes were blocked with 3% bovine serum albumin in Tris-buffered saline containing 0.1% Tween 20 (TBS-T) for 1 hour and then further incubated overnight at 4°C with the following primary antibodies: CD36 (1 : 2000), p-AKT (1 : 1000), AKT (1 : 1000), PPAR*γ* (1 : 1000), and *β*-actin (1 : 2000). Primary antibody against CD36 was purchased from Novus Biologicals, USA. Primary antibodies against p-AKT, AKT, PPAR*γ*, and *β*-actin were purchased from Cell Signaling Technology, USA. After sufficiently washed in TBS-T, the membranes were incubated with horseradish peroxidase-labelled goat anti-rabbit antibodies (1 : 5000, ZSGB-BIO, Beijing, China) for 1 hour at 37°C. Finally, proteins were visualized using ECL Advance western Blotting Detection kit (Amersham Bioscience, Piscataway, USA). Band densities were measured by Image J program and normalized to the band density of the corresponding *β*-actin.

### 2.3. Total RNA Extraction and Real-Time PCR

Total RNA was extracted from cultured HK-2 cells using a RNAiso Kit (Takara, Dalian, China) in accordance with the manufacturer's recommendations. Reverse transcription was performed from 1 *μ*g of total RNA in a 20 *μ*L reaction system using a cDNA synthesis kit (Takara, Dalian, China). The reaction was performed at 37°C for 15 minutes and then at 85°C for 5 minutes. By using SYBR Green dye, real-time PCR was carried out in a real-time PCR machine. The thermal cycling program was 2 min at 50°C for enzyme activation, 40 cycles of denaturation for 20 s at 95°C, 20 s annealing at 55°C, and 30 s extension at 72°C. mRNA values were normalized to *β*-actin mRNA. The specific primers used were human CD36 (forward) 5′-AAATAAACCTCCTTGGCCTGA-3′ and (reverse) 5′-GCAACAAACATCACCACACC-3′ and human *β*-actin (forward) 5′-CCTGGCACCCAGCACAAT-3′ and (reverse) 5′-GCCGATCCACACGGAGTA-3′.

### 2.4. Immunofluorescence

HK-2 cells were treated in six-well chamber slides and fixed with cold acetone for 15 min at 4°C. Then the cells were further incubated with CD36-specific antibody (1 : 100) overnight at 4°C. Next, the HK-2 cells were incubated with a fluorescein isothiocyanate- (FITC-) conjugated goat anti-rabbit secondary antibody (ZSGB-BIO, Beijing, China) at dilutions of 1 : 100 for 60 min at 37°C. Nucleus was counterstained with 4,6-diamidino-2-phenylindole (DAPI, Beyotime Institute of Biotechnology, China) for 15 min. The cells were examined under a fluorescence microscope.

### 2.5. Oil Red O Staining

HK-2 cells were grown and treated in six-well chamber slides. Subsequently, the cells were fixed in 4% paraformaldehyde for 10 min and stained with 0.3% Oil Red O for 30 min. Finally, the cells were counterstained by hematoxylin for 5 min and examined by light microscopy. To quantify the relative levels of lipids, the HK-2 cells were subjected to air-dry for 40 min and the Oil Red O was eluted with 1 mL 100% isopropanol. The eluted dye was aliquoted in individual wells of a 96-well plate to determine absorbance at 500 nm and 100% isopropanol was used as the blank control.

### 2.6. Cell Viability Assay

HK-2 cell viability was detected using the Cell Counting Kit-8 (CCK-8, Yiyuan Biotechnologies, China) in accordance with the manufacturer's instructions. Briefly, the counted HK-2 cells were seeded into a 96-well culture plate and incubated in the experimental media and then stimulated with various treatments as described above. Next, the CCK-8 solutions were added to each well and further incubated with the cells for 2 h at 37°C. Finally, the absorbance was measured at 450 nm using a microplate reader, which could be illustrated, indicating a relative percentage of the viability of counted cells.

### 2.7. Statistical Analysis

All experiments were repeated thrice. All experiment data were expressed as mean ± standard deviations (SD) and analyzed using SPSS 22.0 for Windows. Data were analyzed using unpaired two-tailed Student's *t*-test or ANOVA with Scheffe's multiple testing correction. *P* value < 0.05 was considered statistically significant.

## 3. Results

### 3.1. HG Promotes CD36 Expression in a Time-Dependent Manner, While NG Has No Effect on CD36 Expression in HK-2 Cells

**Figure 1:**
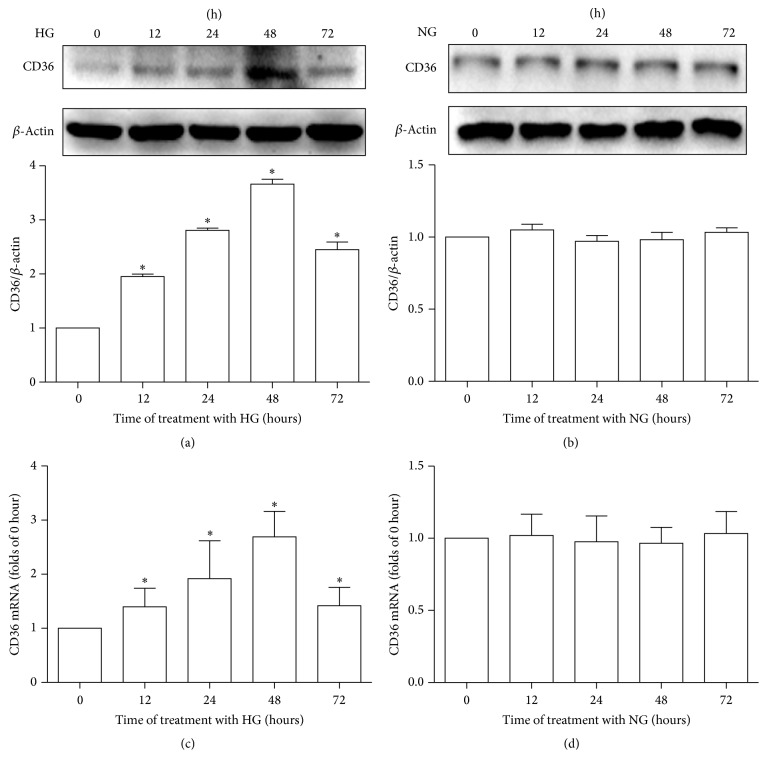
Effects of high glucose or normal glucose on CD36 expression in HK-2 cells. HK-2 cells were treated with high glucose (30 mM, HG) or normal glucose (5.6 mM, NG) for 0 h, 12 h, 24 h, 48 h, or 72 h. The expression of CD36 was examined at the indicated time points in the HK-2 cells. All experiments were repeated thrice. CD36 protein levels were determined by western blotting. Band intensities were normalized to *β*-actin band intensity using densitometry. The data were represented as the means ± SD (a, b). CD36 mRNA levels were measured by RT-qPCR. *β*-Actin served as a reference gene. The data were represented as means ± SD (c, d). ^*∗*^*P* < 0.05 versus control (0 h).

To determine the effects of HG and NG on CD36 expression in vitro, HK-2 cells were treated with HG or NG for the indicated times and then the cellular CD36 expression levels were determined by western blotting and RT-qPCR. As shown in Figure 1, HG induced the expression of CD36 in a time-dependent manner in HK-2 cells. CD36 mRNA and protein expression levels in the HG-treated HK-2 cells started to increase significantly at 12 h after treatment and peaked at 48 h after treatment (Figures 1(a) and 1(c)). However, CD36 mRNA and protein expression levels remained unchanged in the NG-treated HK-2 cells across all time points examined (Figures 1(b) and 1(d)).

### 3.2. Inhibition of AKT Phosphorylation Alleviates HG-Induced Overexpression of CD36 in HK-2 Cells

**Figure 2:**
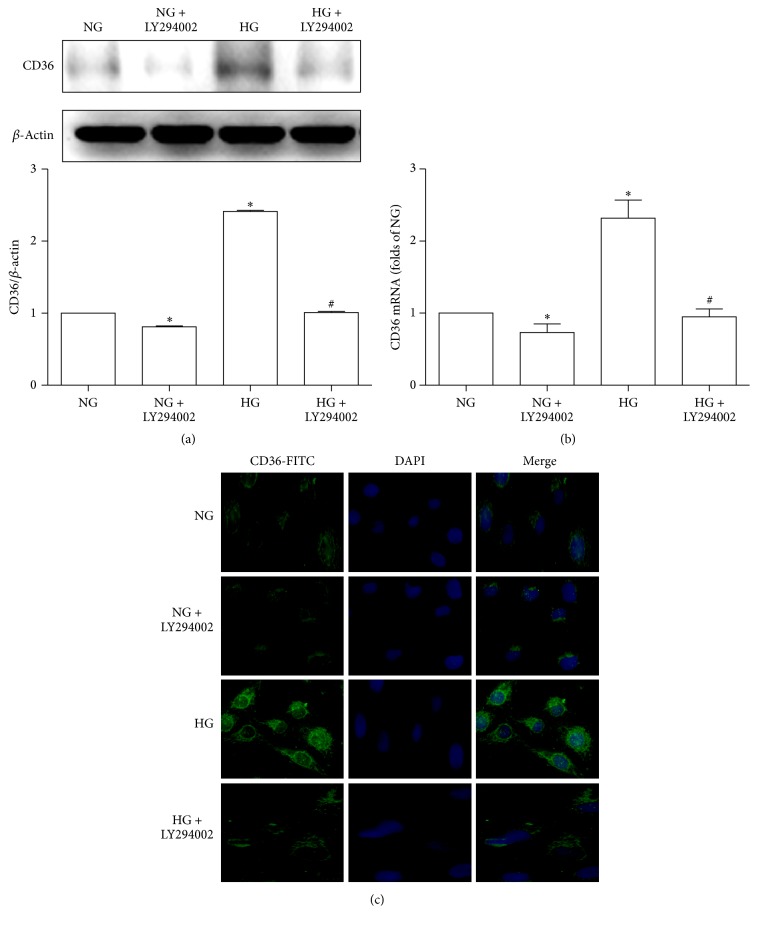
Effects of LY294002 on HG-induced CD36 expression. HK-2 cells were cultured with normal glucose (5.6 mM, NG), NG plus LY294002 (15 uM), high glucose (30 mM, HG), or HG plus LY294002 for 48 h. CD36 protein levels were determined by western blotting. Band intensities were normalized to *β*-actin band intensity using densitometry. The histogram represented the normalized intensities of proteins from three experiments. The data were represented as the means ± SD (a). CD36 mRNA levels were measured by RT-qPCR. *β*-Actin served as a reference gene. The results from three independent experiments were represented as means ± SD (b). ^*∗*^*P* < 0.05 versus NG; ^#^*P* < 0.05 versus HG. Immunofluorescence staining of CD36 was shown in (c).

We examined whether inhibition of AKT pathway could reduce the HG-induced overexpression of CD36 in HK-2 cells. The results showed that HG increased the expressions of CD36 mRNA and protein in HK-2 cells. However, LY294002, a selective AKT inhibitor, decreased the HG-induced CD36 expression in HK-2 cells. Under NG conditions, LY294002 also decreased the expression levels of CD36 in HK-2 cells (Figures 2(a) and 2(b)). Immunofluorescence examination of CD36 confirmed the above findings (Figure 2(c)).

### 3.3. HG Upregulates PPAR*γ* Expression by Activating AKT, Which Can Regulate CD36 Expression in HK-2 Cells

**Figure 3:**
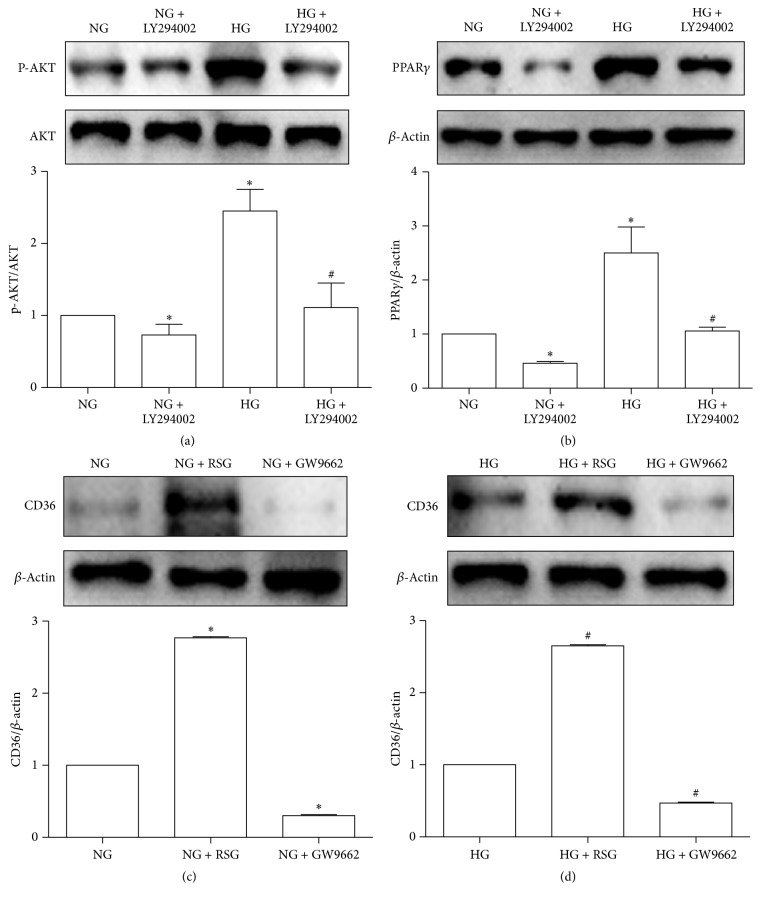
PPAR*γ* is upregulated by the HG-promoted AKT phosphorylation, which can regulate CD36 expression in HK-2 cells. HK-2 cells were cultured with NG (5.6 mM), NG + LY294002 (15 uM), HG (30 mM), or HG + LY294002 (15 uM) for 48 h. P-AKT, AKT, and PPAR*γ* were examined by western blotting (a, b). HK-2 cells were pretreated with an agonist (RSG, 5 uM)/antagonist (GW9662, 2.5 uM) of PPAR*γ* for 1 h, followed by 48 h of NG (5.6 uM)/HG (30 uM) stimulation for the analysis of CD36 protein levels. Using western blotting, cell lysates were analyzed (c, d). All experiments were repeated thrice. Band intensities were normalized to *β*-actin band intensity using densitometry. The data were represented as the means ± SD. ^*∗*^*P* < 0.05 versus NG; ^#^*P* < 0.05 versus HG.

Our results showed that HG upregulated AKT phosphorylation and PPAR*γ* expression and inhibition of AKT phosphorylation by LY294002 could decrease the HG-induced PPAR*γ* expression in the HK-2 cells. Under NG conditions, LY294002 also decreased AKT phosphorylation and PPAR*γ* expression in the HK-2 cells (Figures 3(a) and 3(b)). To further study the role of PPAR*γ* in regulating CD36 expression, we used western blotting to examine the expression levels of CD36 in HK-2 cells treated or not treated with an agonist (RSG)/antagonist (GW9662) of PPAR*γ* under NG and HG conditions. As anticipated, the results showed that the expression levels of CD36 were increased by RSG but decreased by GW9662 in the HK-2 cells under NG and HG conditions (Figures 3(c) and 3(d)). These data suggest that HG can activate AKT-PPAR*γ* signaling pathway in HK-2 cells and PPAR*γ* plays an important role in regulating CD36 expression.

### 3.4. Inhibition of AKT-PPAR*γ* Signaling Pathway or Knockdown of CD36 Reduces HG-Induced Lipid Accumulation in HK-2 Cells

**Figure 4:**
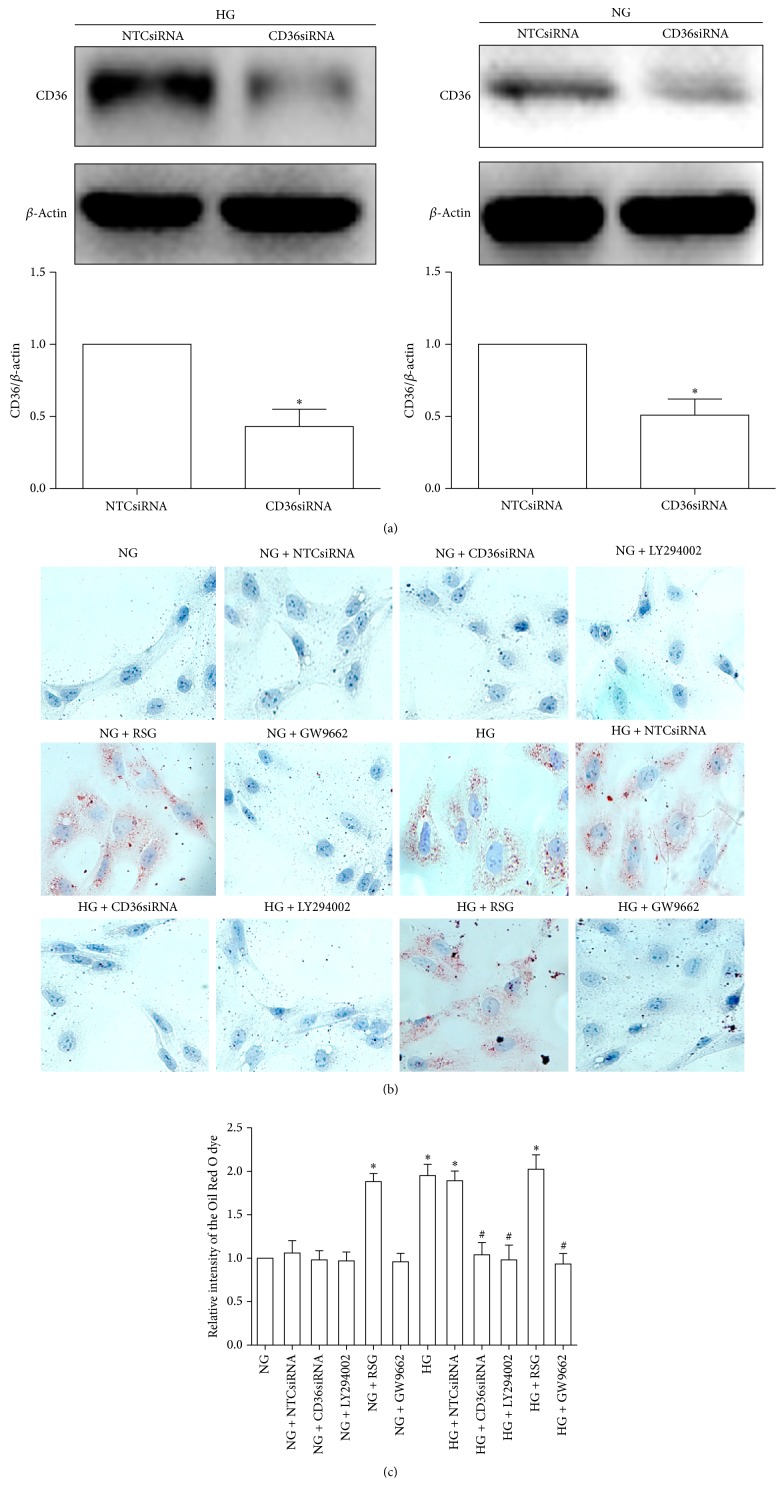
The roles of AKT-PPAR*γ* signaling pathway and CD36 in regulating HG-induced lipid accumulation in HK-2 cells. The HK-2 cells were transfected with CD36siRNA or nontargeting control siRNA (NTC siRNA) under HG (30 uM) and NG (5.6 uM) conditions and western blot analysis was used to determine CD36siRNA-mediated knockdown of CD36 in the HK-2 cells. Band intensities were normalized to *β*-actin band intensity using densitometry. The data from three independent experiments were represented as means ± SD (a). ^*∗*^*P* < 0.05 versus NTCsiRNA. By using Oil Red O staining, lipid content was detected in the HK-2 cells incubated with NG (5.6 mM), NG + NTCsiRNA, NG + CD36siRNA, NG + LY294002 (15 uM), NG + RSG (5 uM), NG + GW9662 (2.5 uM), HG (30 mM), HG + NTCsiRNA, HG + CD36siRNA, HG + LY294002 (15 uM), HG + RSG (5 uM), and HG + GW9662 (2.5 uM) for 48 h, respectively (b). Relative levels of lipid content were quantified by measuring relative absorbance of the eluted Oil Red O dye at 500 nm. Data from three independent experiments were represented as means ± SD (c). ^*∗*^*P* < 0.05 versus NG; ^#^*P* < 0.05 versus HG.

To knockdown significantly CD36 expression, HK-2 cells were stably transfected with CD36siRNA or nontargeting control siRNA (NTCsiRNA) as controls. We could not identify the CD36siRNA-transfected HK-2 cells with 100% knockdown of CD36, perhaps attributing to an essential role of CD36 in cell survival. However, CD36 siRNA reduced the levels of CD36 protein in the HK-2 cells to approximately 50% of controls under NG and HG conditions (Figure 4(a)). In order to identify the roles of AKT-PPAR*γ* signaling pathway and CD36 in regulating HG-induced lipid accumulation, we used Oil Red O staining to detect lipid content in the HK-2 cells. The Oil Red O staining results indicated that HG induced lipid deposition in the HK-2 cells, which could be alleviated by CD36siRNA, LY294002, or GW9662. Under NG conditions, RSG also induced lipid deposition in the HK-2 cells (Figure 4(b)). Furthermore, the relative levels of eluted Oil Red O dye were quantified. We found that the dye levels in the HG-treated HK-2 cells were higher than that in the NG-treated HK-2 cells. However, the dye levels in the HG-treated HK-2 cells could be decreased by CD36siRNA, LY294002, or GW9662. Additionally, RSG treatment also led to an increase in dye levels in the HK-2 cells under NG conditions (Figure 4(c)). These results suggest that AKT-PPAR*γ* signaling pathway and CD36 are involved in HG-induced lipid deposition in HK-2 cells.

### 3.5. Inhibition of AKT-PPAR*γ* Signaling Pathway or Knockdown of CD36 Reverses HG-Induced Decrease in Cell Viability in HK-2 Cells

**Figure 5:**
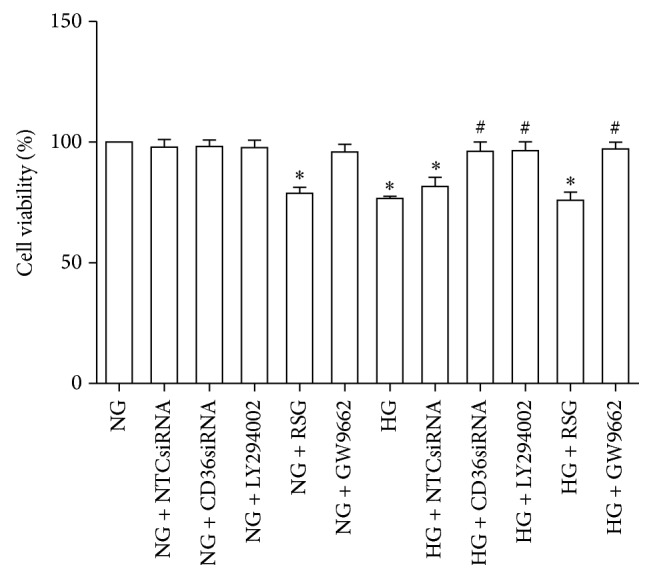
Regulation of HK-2 cell viability by CD36siRNA, LY294002, RSG, or GW9662 under NG and HG conditions. The HK-2 cells were incubated with NG (5.6 mM), NG + NTCsiRNA, NG + CD36siRNA, NG + LY294002 (15 uM), NG + RSG (5 uM), NG + GW9662 (2.5 uM), HG (30 mM), HG + NTCsiRNA, HG + CD36siRNA, HG + LY294002 (15 uM), HG + RSG (5 uM), or HG + GW9662 (2.5 uM) for 48 h. Cell viability was determined by using CCK-8 assay. Data from three independent experiments were expressed as means ± SD. ^*∗*^*P* < 0.05 versus NG; ^#^*P* < 0.05 versus HG.

HK-2 cell viability was evaluated using CCK-8 assay. Compared with the NG-treated HK-2 cells, the HG-treated HK-2 cells showed a decrease in cell viability. Such decrease was attenuated in the HK-2 cells treated by CD36siRNA, LY294002, or GW9662. Additionally, RSG treatment also led to a decrease in cell viability in the HK-2 cells under NG conditions (Figure 5).

## 4. Discussion

In the current study, we aimed to examine and identify the mechanisms of HG-induced overexpression of CD36 in renal tubular cells. Here we first propose that HG promotes CD36 expression through the activation of AKT-PPAR*γ* signaling pathway, leading to lipid deposition in renal tubular cells. Previous studies have demonstrated that HG induces CD36 overexpression in diabetic kidney, which plays various roles in the pathogenesis of DKD [30]. As a multifunctional protein, CD36 plays an important role in the storage of fatty acids and in the provision of fatty acids that synthesize adenosine triphosphate (ATP) by an energy-yielding pathway defined as *β*-oxidation [31, 32]. HG-induced CD36 overexpression may increase the cellular uptake of free fatty acids and result in higher intracellular lipid accumulation in diabetic kidney. However, the precise mechanisms by which HG causes CD36 overexpression are not completely understood. Our study not only demonstrated that HG induced lipid deposition through the increased CD36, but also explored the molecular mechanisms by which HG upregulated CD36 expression in renal tubular cells. Our data therefore suggested a mechanistic role for AKT-PPAR*γ* signaling pathway by which HG induced CD36 overexpression in renal tubule epithelial cells.

We found that HG upregulated CD36 mRNA and protein expression in a time-dependent manner in cultured HK-2 cells. This result is in line with a study showing that hyperglycemia promotes CD36 mRNA and protein expression selectively in proximal tubules of human diabetic kidney [23]. DKD is strongly related to hyperglycemia and lipid disorder characterized by an increased intracellular and plasma fatty acid availability. As a fatty acid translocase, CD36 is logically upregulated in HG-treated HK-2 cells, which contributes to uptake and intracellular transport of free fatty acids.

Whether activation of AKT signaling pathway is implicated in CD36 expression in HG-treated renal tubular cells has not been proved. To explore the role of AKT signaling pathway, AKT phosphorylation was inhibited by the use of chemical inhibitor LY294002. Our results in vitro indicated that the HG-induced upregulations of CD36 mRNA and protein expression were markedly abrogated by LY294002. The results suggested that AKT signaling pathway played an important role in mediating HG-induced CD36 expression in HK-2 cells. Considering that PPAR*γ* is a key transcription factor upregulating CD36 expression in many different types of cells, we have investigated whether HG promotes CD36 expression via activating AKT-PPAR*γ* signaling pathway in HK-2 cells. Firstly we explored whether HG promoted AKT phosphorylation in HK-2 cells. Our results indicated that the levels of phospho-AKT were greatly elevated among the HK-2 cells stimulated by HG. To further investigate the role of AKT phosphorylation in mediating HG-induced PPAR*γ* expression, we examined the levels of PPAR*γ* expression in the HK-2 cells treated with or without LY294002 under NG and HG conditions. PPAR*γ* expression was significantly elevated in the HG-treated HK-2 cells compared with the NG-treated HK-2 cells. However, LY294002 attenuated the HG-induced increase in PPAR*γ* expression, which meant AKT phosphorylation mediated HG-induced PPAR*γ* expression in HK-2 cells. To demonstrate a direct role for PPAR*γ* in regulating CD36 expression, we examined the expression levels of CD36 in the HK-2 cells treated with an agonist (RSG)/antagonist (GW9662) of PPAR*γ* under NG and HG conditions. As anticipated, the expression of CD36 was increased in the HK-2 cells treated with RSG but decreased in the HK-2 cells treated with GW9662 under NG and HG conditions. These data showed that upregulation of CD36 by HG required AKT-PPAR*γ* signaling pathway in HK-2 cells.

Our study also sought to determine whether HG caused lipid deposition in renal tubular cells by upregulating CD36 expression. By using Oil Red O staining and quantifying the dye eluted from stained cells, we confirmed that HG caused lipid deposition in HK-2 cells, consistent with the previous studies [33, 34]. Additionally, RSG, an agonist of PPAR*γ*, which could promote CD36 expression via PPAR*γ*, also had a similar effect of inducing lipid deposition in HK-2 cells under NG conditions. Further, we found that inhibition of AKT-PPAR*γ* signaling pathway or knockdown of CD36 obviously attenuated lipid deposition in the HG-treated HK-2 cells. These findings, in combination with the result that HG upregulates CD36 expression via AKT-PPAR*γ* signaling pathway, suggest a role for CD36 in mediating HG-induced lipid deposition in HK-2 cells.

It is well known that lipid accumulation plays a critical role in the cellular mechanism of cell death in many human diseases, including diabetes, Parkinson's disease, and atherosclerosis [35–37]. Previous studies have demonstrated that intracellular lipid accumulation can trigger endoplasmic reticulum stress (ERS), increase reactive oxygen species (ROS) production, and induce inflammatory responses, eventually leading to cell death [38–40]. Therefore, the final experiment was performed to show the association between lipid accumulation and cell viability in HK-2 cells. We found that the indicated treatments responsible for intracellular lipid accumulation also reduced cell viability in HK-2 cells. Taken together, increased CD36 expression can upregulate free fatty acids uptake and then induce intracellular lipid accumulation, reducing cell viability in HK-2 cells under HG conditions.

Our findings have practical clinical importance. Treatment aimed at AKT-PPAR*γ* pathway may attenuate HG-induced lipid accumulation by downregulating CD36 expression in diabetic kidney. However, AKT signaling pathway can also regulate other downstream targets including Forkhead box protein O1 (FoxO1), mammalian target of rapamycin (mTOR), and proline-rich AKT substrate 40 kDa (PRAS40). Therefore, further investigations are necessary to determine whether other pathways are involved in AKT-mediated upregulation of CD36.

In summary, this study shows that HG promotes CD36 expression by upregulating AKT-PPAR*γ* signaling pathway, resulting in lipid deposition and decreased cell viability in renal tubular cells. AKT-PPAR*γ* signaling pathway is a potential therapeutic target for treating lipid deposition in DKD.
